# Associations of Depressive Symptom Severity with High-Sensitivity C-Reactive Protein Among U.S. Adults: NHANES 2015–2018

**DOI:** 10.3390/jcm15082975

**Published:** 2026-04-14

**Authors:** Diego Rivera-Porras, Daniel Cepeda-Pineda, Sandra-Milena Carrillo-Sierra, Omar Rozo-Pérez, Astrid Rozo-Sánchez, Valmore Bermúdez

**Affiliations:** 1 Universidad de la Costa, Departamento de Productividad e Innovación, Barranquilla 080001, Atlántico, Colombia; 2 Universidad de la Sabana, Facultad de Ciencias del Comportamiento, Chía 250007, Cundinamarca, Colombia; danielcepi@unisabana.edu.co; 3 Universidad de Pamplona, Facultad de Psicología, Grupo de Investigación Psicología y Sociedad, Pamplona 543050, Norte de Santander, Colombia; sandra.carrillo3@unipamplona.edu.co; 4 Universidad Simón Bolívar, Facultad de Ciencias Jurídicas y Sociales, Centro de Investigación en Estudios Fronterizos, Cúcuta 540001, Norte de Santander, Colombia; 5 Universidad de Pamplona, Facultad de Salud, Pamplona 543050, Norte de Santander, Colombia; astrid.rozo@unipamplona.edu.co; 6 Universidad Simón Bolívar, Centro de Investigaciones en Ciencias de la Vida, Barranquilla 080001, Atlántico, Colombia; valmore.bermudez@unisimon.edu.co

**Keywords:** NHANES, PHQ-9, depressive symptoms, high-sensitivity C-reactive protein, inflammation, prevalence ratio, survey-weighted analysis, biomarkers, U.S. adults, mental health

## Abstract

**Background:** Depressive symptoms have been linked to systemic inflammation, yet estimates in population-representative data vary by symptom severity and analytic specifications. We quantified the association between depressive symptom severity and high-sensitivity C-reactive protein (hs-CRP) in U.S. adults using design-based inference. **Methods:** We analysed pooled NHANES 2015–2018 data for adults aged ≥ 20 years (unweighted *n* = 9164; complete-case adjusted models *n* = 8173). Depressive symptom severity was categorised using the Patient Health Questionnaire-9 (PHQ-9) with 0–4 as the reference group and a pre-specified primary contrast of 10–14 versus 0–4. Outcomes were (i) continuous hs-CRP modelled on the log scale, reported as geometric mean ratios (GMR), and (ii) elevated inflammation defined as hs-CRP > 3 mg/L, modelled using a log-link to obtain prevalence ratios (PR). Models incorporated NHANES complex sampling and adjusted for a pre-specified core covariate set (age, sex, race/ethnicity, education, poverty-income ratio, and smoking). Sensitivity analyses excluded hs-CRP > 10 mg/L and added BMI. **Results:** After adjustment, the geometric mean hs-CRP was 1.43 mg/L (95% CI 1.21–1.70) for PHQ-9 0–4 and 1.63 mg/L (95% CI 1.29–2.08) for PHQ-9 10–14. For the primary contrast (10–14 vs. 0–4), the adjusted GMR was 1.14 (0.96–1.35) and the PR was 1.15 (0.95–1.39). Using a clinically relevant dichotomy (PHQ-9 ≥ 10 vs. <10), depressive symptoms were associated with higher hs-CRP (GMR 1.24 (1.07–1.43)) and a higher prevalence of hs-CRP > 3 mg/L (PR 1.19 (1.01–1.39)). Associations were strongest for PHQ-9 15–19 (GMR 1.62 (1.20–2.19); PR 1.49 (1.15–1.92)). In sensitivity analyses for the primary contrast, GMR estimates ranged from 1.01 to 1.14 and PR estimates ranged from 1.05 to 1.15, with attenuation towards the null after excluding hs-CRP > 10 mg/L and after additional adjustment for BMI. **Conclusions:** Higher depressive symptom severity was associated with higher hs-CRP and a higher prevalence of low-grade systemic inflammation in U.S. adults, with the clearest elevations observed among those with moderately severe symptoms. For the pre-specified moderate-symptom contrast, point estimates were modest and sensitive to handling of high hs-CRP values and adiposity-related adjustment.

## 1. Introduction

Depression is a leading contributor to disability worldwide and remains a major public health priority across the life course. Current estimates indicate that depression affects around 280 million people globally (approximately 3.8% of the population), with substantial impacts on functioning, quality of life, and health service demand [1].

Beyond its chronic burden, depression prevalence and severity can shift in response to population-level stressors. Using Global Burden of Disease methods, the COVID-19 pandemic period was associated with an estimated 27.6% increase in major depressive disorder in 2020 (tens of millions of additional cases worldwide), underscoring the sensitivity of depressive morbidity to social disruption and stressor exposure [2].

In the United States, nationally representative data also indicate a non-trivial baseline burden. Analyses of National Center for Health Statistics survey data reported that approximately 8.1% of adults had depression in 2013–2016, with higher prevalence among women than men and gradients by age and socioeconomic indicators [3]. These patterns motivate continued attention to pathways that link depressive symptomatology with downstream health risk.

Cardiometabolic and cardiovascular outcomes are among the most consequential clinical correlates of depression. Meta-analytic evidence from prospective cohorts has reported an increased risk of coronary heart disease among individuals with depression [4]. In parallel, evidence syntheses in cardiovascular populations indicate that depressive disorders and symptoms are common, with pooled prevalence estimates around one-fifth in some analyses [5]. Together, these findings support the view that depression is embedded within broader risk profiles that may accumulate over time.

Inflammation has been proposed as one plausible biological pathway linking depression with somatic morbidity. The ‘sickness behaviour’ framework describes a coordinated behavioural and affective response to immune activation-characterised by fatigue, anhedonia, and reduced social engagement-that overlaps phenomenologically with depressive symptoms and is linked to inflammatory signalling [6].

Contemporary psychoneuroimmunology further emphasises that stress exposure can initiate and sustain inflammatory processes via neuroendocrine and autonomic pathways, with downstream effects on brain circuits relevant to mood regulation. A social signal transduction account highlights how social threat, adversity, and chronic stress can amplify inflammatory tone and thereby increase vulnerability to depressive symptoms in susceptible individuals [7].

Inflammation in depression is unlikely to be monolithic. Reviews integrating clinical and experimental evidence point to heterogeneity in inflammatory biomarkers across patients, with a subset exhibiting elevated inflammatory markers and potentially distinct clinical features or treatment response profiles [8]. This heterogeneity is clinically important because it implies that average associations may obscure clinically meaningful subgroup structure.

At a mechanistic level, several immune-to-brain pathways have been implicated. These include cytokine-mediated effects on monoaminergic transmission, neuroplasticity, and reward processing, as well as microglial activation and alterations in glutamatergic signalling. Inflammasome-related pathways have been proposed as a bridging mechanism connecting psychological stressors, innate immune activation, and depressive symptom expression [9].

C-reactive protein (CRP), particularly when measured using high-sensitivity assays (hs-CRP), is a widely used marker of systemic low-grade inflammation. hs-CRP is routinely used in cardiovascular risk assessment, and recent clinical guidance incorporates hs-CRP (for example, ≥2 mg/L) as a ‘risk-enhancing’ factor in selected contexts, reflecting its prognostic value for atherosclerotic events [10].

Empirically, inflammatory markers have been linked to depressive symptoms across observational and experimental paradigms. Syntheses of the literature emphasise that inflammatory activation is not uniform across patients and that associations vary by design, population, and symptom profile, reinforcing the rationale for population-based analyses that characterise graded relations between depressive symptom severity and hs-CRP [11].

Interpretation is complicated by confounding and reverse causation. Depressive symptoms co-occur with behaviours and conditions that influence inflammation (e.g., smoking, sleep disruption, and metabolic disease), while inflammatory processes may also precede or intensify specific symptom clusters. Evidence from large biobank samples suggests that individuals with depression can show higher CRP concentrations and that inflammatory status can track with symptom burden and comorbidity patterns [12].

Clinical implications also extend to treatment stratification. A systematic review and meta-analysis of randomised antidepressant trials has suggested that baseline inflammatory biomarkers may index differential treatment response, consistent with a partially distinct inflammatory endophenotype for a subset of patients [13]. Population-based evidence on hs-CRP gradients across depressive symptom severity may therefore inform etiological understanding as well as potential stratification hypotheses.

Symptom-level approaches have gained traction because depression is phenotypically heterogeneous. Network-oriented analyses indicate that systemic low-grade inflammation may align more strongly with somatic or neurovegetative symptoms (e.g., sleep and energy) than with cognitive-affective symptoms, implying that aggregate depression scores may obscure specific inflammatory correlates [14]. Clinical studies likewise suggest that CRP can relate to overall symptom severity and particular symptom domains in major depressive disorder [15].

Obesity and related metabolic dysregulation are particularly salient because they are strongly related to both inflammation and depression. Conceptual and empirical work supports an ‘immunometabolic’ phenotype in which adiposity-related inflammatory activity contributes to depressive symptom expression and comorbidity patterns [16]. Accordingly, careful handling of adiposity and metabolic covariates is essential when estimating associations between depressive symptoms and hs-CRP.

Cross-national population studies illustrate the complexity of these interrelations. Analyses from the Korea National Health and Nutrition Examination Survey reported sex differences in the association between hs-CRP and depression [17], while German nationally representative data suggested that obesity and depressive symptoms may jointly relate to CRP [18]. Such findings motivate analyses that consider effect heterogeneity and symptom structure rather than relying solely on single summary scores.

In U.S. NHANES data, multiple analyses have examined the link between depressive symptoms and hs-CRP, with stronger associations often observed at higher symptom burden and attenuation after adiposity-related adjustment. For example, in NHANES 2015–2018, a recent population-based study assessed body mass index (BMI)-related effect modification and noted that estimates were sensitive to BMI adjustment, particularly in moderate symptom strata [19]. Other nationally representative analyses have likewise highlighted interrelations among depressive symptoms, adiposity, and systemic inflammation, although differences in outcome specification and effect measures (e.g., continuous vs. threshold hs-CRP, odds ratios vs. prevalence ratios) complicate cross-study comparability [20].

Accordingly, the incremental contribution of the present analysis is not to re-establish the existence of a depression-inflammation association, but to provide severity-graded, design-consistent estimates using contemporary NHANES cycles while jointly reporting both continuous and clinically interpretable hs-CRP outcomes. Specifically, we estimate (i) geometric mean ratios for continuous hs-CRP and (ii) prevalence ratios for hs-CRP > 3 mg/L across PHQ-9 categories, prioritising a pre-specified moderate-symptom contrast (PHQ-9 10–14 vs. 0–4) to balance clinical meaning and statistical precision [11,19].

We further evaluate robustness to alternative modelling choices, including exclusion of hs-CRP > 10 mg/L to reduce influence of acute-phase responses and additional adjustment for BMI. Finally, motivated by prior population evidence of sex differences in depression-inflammation associations [17], we examine effect heterogeneity by sex in exploratory interaction models and use natural spline models to avoid assuming linearity across the PHQ-9 score range.

Research questions: (1) Among U.S adults, what is the association between depressive symptom severity and hs-CRP as a continuous biomarker of systemic inflammation? (2) Among U.S adults, what is the association between depressive symptom severity and the prevalence of elevated hs-CRP (using clinically motivated thresholds for higher inflammatory risk)? (3) Do these associations vary by sex?

Objective: To estimate the association between depressive symptom severity and systemic inflammation measured by hs-CRP in a nationally representative sample of U.S adults. Specific objectives were to (i) quantify adjusted differences in hs-CRP across depressive symptom severity categories and for a clinically relevant PHQ-9 cut-point (≥10 vs. <10), (ii) estimate adjusted prevalence ratios for elevated hs-CRP (>3 mg/L) across depressive symptom severity categories, (iii) evaluate robustness of these associations to alternative modelling choices (including exclusion of hs-CRP > 10 mg/L and additional adjustment for BMI), and (iv) explore effect heterogeneity by sex.

## 2. Materials and Methods

### 2.1. Study Design and Data Source

We conducted a cross-sectional, design-based analysis using data from the National Health and Nutrition Examination Survey (NHANES). NHANES is a continuous, nationally representative survey of the civilian, non-institutionalised U.S population, based on a complex, multistage probability design with stratification and clustering. Two continuous cycles (2015–2016 and 2017–2018) were pooled, following official analytic guidance for combining cycles in NHANES [21,22,23]. The objective was to estimate adjusted associations (not causal effects) between depressive symptom severity and systemic inflammation measured by high-sensitivity C-reactive protein (hs-CRP).

### 2.2. Study Population and Eligibility Criteria

The pooled NHANES 2015–2018 sample included 19,225 participants. We restricted the analytic population to adults aged ≥ 20 years who attended the Mobile Examination Center (MEC) examination (*n* = 11,288), because MEC examination weights are required for nationally representative inference of laboratory measures [21,22,23]. Among eligible adults, hs-CRP was missing for 1246 participants (11.0%), leaving 10,042 with laboratory measurement. Of those, PHQ-9 was incomplete for 878 participants (8.7%), yielding an analytic pool of 9164 with complete exposure and outcome for descriptive analyses. Primary adjusted models additionally required complete data for the core covariate set (age, sex, race/ethnicity, education, poverty-income ratio, and smoking), resulting in a complete-case sample of *n* = 8173 (exclusions due to missing core covariates: 991/9164 (10.8%), primarily missing PIR (*n* = 987; 10.8%)). In sensitivity analyses, excluding hs-CRP > 10 mg/L removed 721/8173 participants (8.8%), yielding *n =* 7452. BMI-adjusted sensitivity models were estimated among participants with non-missing BMI (*n* = 8099; 74/8173 (0.9%) missing).

### 2.3. Exposure Assessment: Depressive Symptoms (PHQ-9)

Depressive symptoms were assessed using the PHQ-9, a brief instrument that captures the frequency of nine depressive symptoms over the prior two weeks. Each item is scored from 0 (not at all) to 3 (nearly every day), yielding a total score ranging from 0 to 27 [24,25]. The PHQ-9 is widely used in clinical and epidemiological research and has demonstrated acceptable psychometric properties for assessing depressive symptom severity [24]. The primary exposure specification categorised the PHQ-9 total score into standard severity groups: 0–4 (minimal), 5–9 (mild), 10–14 (moderate), 15–19 (moderately severe), and 20–27 (severe) [24]. The pre-specified primary contrast compared PHQ-9 10–14 versus 0–4, reflecting clinically meaningful symptoms relative to minimal symptomatology. As a clinically oriented sensitivity analysis, we additionally evaluated a dichotomous exposure (PHQ-9 ≥ 10 versus < 10) [24].

### 2.4. Outcome Assessment: hs-CRP (Continuous and Clinical Threshold)

The primary outcome was serum hs-CRP concentration (mg/L) measured from venous blood specimens collected during the MEC examination, processed, and shipped frozen to central laboratories using standardised NHANES protocols [26,27]. In 2015–2016, hs-CRP was quantified on the Beckman Coulter UniCel DxC 600/660i system; in 2017–2018, hs-CRP was quantified on the Roche Cobas 6000 system [26,27]. NHANES flags results below the assay lower limit of detection (LLOD) using a laboratory comment code and imputes these values as LLOD/√2 in the public-release files; LLOD was 0.11 mg/L in 2015–2016 and 0.15 mg/L in 2017–2018 [26,27]. Because hs-CRP is right-skewed, the main analyses treated hs-CRP as a continuous variable on the natural logarithmic scale. In survey-weighted generalised linear models, coefficients for PHQ-9 categories were exponentiated to yield geometric mean ratios (GMR) relative to the reference category (PHQ-9 0–4). A secondary binary outcome defined elevated inflammation as hs-CRP > 3 mg/L, a commonly used threshold in cardiovascular risk contexts for stable individuals [28]. To reduce potential influence of acute inflammatory states, we pre-specified a sensitivity analysis excluding participants with hs-CRP > 10 mg/L [28].

### 2.5. Covariates and Operational Definitions

**Figure 1:**
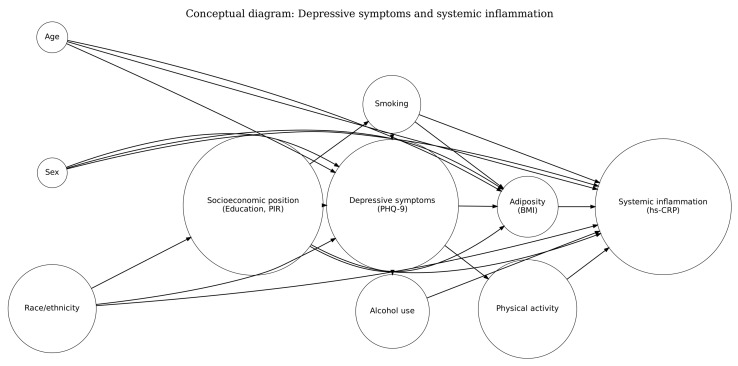
Directed acyclic graph (DAG) for covariate selection. Note: The DAG encodes assumed relationships among depressive symptoms (PHQ-9), hs-CRP, and key sociodemographic and behavioural covariates; arrows indicate assumed causal direction. The core covariate set corresponds to a minimal sufficient adjustment set under d-separation (backdoor criterion) for the PHQ-9-hs-CRP association, blocking non-causal paths while avoiding conditioning on likely downstream variables.

Covariates were selected prior to inferential analyses, informed by prior evidence on depression-inflammation associations and by an explicit conceptual model (directed acyclic graph, DAG) [11,19,20,28]. Using d-separation (backdoor criterion) on the DAG, we identified a minimal sufficient adjustment set for estimating the association between PHQ-9 and hs-CRP. This core adjustment set included sociodemographic and behavioural characteristics plausibly related to both depressive symptoms and systemic inflammation, and not assumed to lie downstream of depressive symptoms: age (years, continuous), sex, race/ethnicity (NHANES categories), educational attainment (NHANES categories), family poverty-income ratio (PIR, continuous), and smoking status derived from standard NHANES smoking items [21,22,23]. These covariates were used consistently in the primary models to address structural confounding while avoiding conditioning on likely downstream variables. The DAG is shown in Figure 1, with arrows indicating assumed causal directions. Body mass index (BMI; kg/m^2^) was reserved for a pre-specified sensitivity analysis because its role may be ambiguous (confounder, mediator, or collider) depending on the assumed causal structure; therefore, BMI was not included in the core adjustment set but was added in alternative models to evaluate robustness [21]. Additional lifestyle variables (e.g., alcohol use and physical activity) were considered as exploratory sensitivities contingent on consistent harmonisation across cycles. The conceptual framework and minimal sufficient adjustment set (DAG) are shown in Figure 1.

The DAG (Figure 1) was used to formalise assumptions about confounding, apply d-separation to define the minimal sufficient adjustment set, and distinguish core adjustment covariates from variables reserved for sensitivity analyses. All results are interpreted as adjusted associations without causal attribution. Any expansion of the adjustment set is documented as sensitivity analysis with explicit reporting of changes in magnitude and precision.

### 2.6. Survey Design Specification and Weighting

All estimates incorporated the NHANES complex survey design using SDMVSTRA (strata) and SDMVPSU (primary sampling units), together with MEC examination weights [21,22,23]. For pooled 2015–2018 analyses, a 4-year MEC weight was constructed by dividing the 2-year MEC weight by two (WTMEC4YR = WTMEC2YR/2), consistent with NHANES guidance for combining two survey cycles [22,23]. Standard errors and 95% confidence intervals were obtained using Taylor series linearisation as implemented in survey analysis tools in R [29].

### 2.7. Statistical Analysis (Estimands, Models, and Reporting)

Descriptive characteristics were summarised using survey-weighted means (with 95% confidence intervals) for continuous variables and survey-weighted percentages for categorical variables, overall and by PHQ-9 category. The primary inferential analysis estimated the association between PHQ-9 categories and continuous hs-CRP (log-transformed) using survey-weighted linear regression for log(hs-CRP) (Gaussian family with identity link) and adjustment for the core covariate set. Exponentiated coefficients were reported as GMR (with 95% confidence intervals), interpreted as the ratio of geometric mean hs-CRP in each PHQ-9 category relative to PHQ-9 0–4 [29]. For the binary outcome hs-CRP > 3 mg/L, prevalence ratios (PR) were estimated using survey-weighted generalised linear models with a log link and a quasi-Poisson working model (svyglm with family = quasipoisson(link = “log”)), with design-based standard errors obtained via Taylor series linearisation [30]. Consistency across the continuous and binary outcomes was evaluated by comparing direction and gradients across PHQ-9 categories. Effect heterogeneity by sex was evaluated in exploratory interaction models including PHQ-9-by-sex terms, summarised using Wald tests.

To explore potential non-linearity, we fitted an alternative model using a natural cubic spline for continuous PHQ-9 total score with 4 degrees of freedom (equivalent to 3 interior knots placed at default quantiles) within the log(hs-CRP) model and generated model-based predictions of geometric mean hs-CRP across the PHQ-9 range. Predictions were evaluated at representative covariate values (median age and median PIR) and fixed categorical covariates (female; never smoker; race/ethnicity and education set to model reference levels) [29]. This analysis was considered exploratory and was reported to support interpretation of dose-response patterns without causal overstatement.

### 2.8. Sensitivity Analyses

Pre-specified sensitivity analyses evaluated: (i) exclusion of hs-CRP > 10 mg/L; (ii) additional adjustment for BMI; (iii) clinical dichotomisation PHQ-9 ≥ 10 versus < 10; and (iv) consideration of potential cycle-related laboratory method differences based on official documentation and pooling guidance [22,23,27]. Sensitivity was summarised by reporting the range of key estimands (GMR and PR for the primary contrast) across these model specifications.

### 2.9. Missing Data

Primary adjusted analyses used a complete-case approach for the core covariate set, yielding *n* = 8173. In the adult MEC pool (*n* = 11,288), missingness was 11.0% for hs-CRP (*n* = 1246) and 14.0% for PHQ-9 total score (*n* = 1575). In the analytic pool with complete exposure and ouUStcome (*n* = 9164), exclusions due to missing core covariates were 991 participants (10.8%), predominantly due to PIR missingness (*n* = 987; 10.8%). Missingness was summarised using variable-level tables and stage-specific attrition summaries, and we assessed whether exclusions due to missingness materially altered key weighted sociodemographic distributions. Because exclusions were primarily driven by missing poverty-income ratio and did not materially change weighted distributions of age, sex, and race/ethnicity, we did not implement multiple imputation in the primary analyses [21,22,23].

### 2.10. Ethics

NHANES procedures and protocols were approved by the National Center for Health Statistics (NCHS) Research Ethics Review Board (ERB), and informed consent procedures are implemented for all participants (written consent for adults; parental permission and assent for minors when applicable) [31]. This study is a secondary analysis of publicly available, de-identified NHANES data without access to identifiable information; therefore, no additional ethical approval was required.

### 2.11. Software, Reproducibility, and Quality Control

Analyses were conducted in R using the survey package for design-based inference; splines for natural spline terms; and emmeans for adjusted geometric means. A script-based reproducible workflow generated analytic datasets, fitted primary and sensitivity models, and produced all tables and figures [29].

## 3. Results

### 3.1. Analytic Sample and Distribution of Depressive Symptom Severity

The analytic pool included 9164 adults aged ≥20 years with complete PHQ-9 data and a valid hs-CRP laboratory measurement, corresponding to 89.0% of adults with MEC examination having hs-CRP available (10,042/11,288) and 91.3% of those having complete PHQ-9 (9164/10,042). Primary adjusted models used the complete-case core covariate sample (*n* = 8173; 89.2% of the analytic pool). Across pooled 2015–2018 cycles, the overall unadjusted geometric mean hs-CRP in the analytic sample was 1.84 mg/L (95% CI 1.73–1.96). Depressive symptom severity was operationalised using standard PHQ-9 categories, with PHQ-9 0–4 as the reference group.

Table 1 provides unweighted counts by PHQ-9 category to contextualise the precision of category-specific estimates. The distribution was highly concentrated in the minimal symptom category (PHQ-9 0–4), with progressively smaller cell sizes in higher PHQ-9 strata. In design-based inference, smaller categories increase sampling variability and inflate standard errors. This becomes particularly relevant for binary outcomes defined by thresholds (here hs-CRP > 3 mg/L), where the effective number of events may be considerably smaller than the total category size. Consequently, interpretation of category-specific associations relies on both the point estimate and the width of the 95% confidence interval.

The pre-specified primary inferential contrast (PHQ-9 10–14 versus 0–4) represented a clinically meaningful difference while retaining a category size compatible with stable estimation. In contrast, the highest symptom category (PHQ-9 20–27) contained relatively few observations, increasing uncertainty for both continuous and binary outcomes. This context is essential for interpreting apparent non-monotonicity across categories, as limited precision can produce overlapping intervals and less stable estimates in the most severe strata.

### 3.2. Descriptive Covariate Patterns by PHQ-9 Category

To contextualise potential confounding, we summarised key sociodemographic and behavioural characteristics across PHQ-9 categories. Table 2 reports survey-weighted means (95% CI) for age, poverty-income ratio (PIR), and BMI and survey-weighted percentages for sex, race/ethnicity, educational attainment, and smoking status. These descriptive gradients do not represent causal effects; rather, they motivate the core adjustment set and help interpret patterns of attenuation in sensitivity analyses.

Higher PHQ-9 categories were characterised by a larger proportion of female participants, substantially higher prevalence of current smoking, and lower socioeconomic position (lower PIR and educational attainment). For example, mean PIR decreased from 3.24 in PHQ-9 0–4 to 1.71 in PHQ-9 20–27, and the proportion college graduate+ decreased from 35.4% to 9.4% across the same contrast. Current smoking increased from 14.1% in PHQ-9 0–4 to 52.7% in PHQ-9 20–27. Mean BMI was higher in PHQ-9 15–19 (31.9 kg/m^2^) than in PHQ-9 0–4 (29.4 kg/m^2^), consistent with adiposity-related confounding or mediation in depression-inflammation associations.

Notably, the smoking distribution also implies potential heterogeneity in inflammatory burden within PHQ-9 strata. Because hs-CRP reflects both chronic low-grade inflammation and acute-phase responses, and because smoking can affect both baseline levels and the probability of exceeding clinical thresholds, differences in smoking prevalence can contribute to differences in both the continuous outcome (geometric mean hs-CRP) and the binary outcome (hs-CRP > 3 mg/L).

### 3.3. Adjusted hs-CRP Levels Across PHQ-9 Categories

Adjusted hs-CRP levels by PHQ-9 category are summarised in Table 3. Estimates are derived from the primary survey-weighted log(hs-CRP) model with the core covariate adjustment set (age, sex, race/ethnicity, education, PIR, and smoking). Results are presented as adjusted geometric means of hs-CRP (mg/L) with 95% confidence intervals.

After core covariate adjustment, hs-CRP levels were higher in categories above the reference group. The adjusted geometric mean in PHQ-9 0–4 was 1.43 mg/L (95% CI 1.21–1.70). Adjusted geometric means were 1.58 mg/L (95% CI 1.29–1.92) for PHQ-9 5–9 and 1.63 mg/L (95% CI 1.29–2.08) for PHQ-9 10–14. The highest adjusted geometric mean occurred in PHQ-9 15–19 (2.32 mg/L; 95% CI 1.59–3.37), followed by PHQ-9 20–27 (1.92 mg/L; 95% CI 1.42–2.60).

In absolute terms, compared with the PHQ-9 0–4 reference group, adjusted geometric mean differences were 5–9: +0.15 mg/L; 10–14: +0.20 mg/L; 15–19: +0.89 mg/L; 20–27: +0.49 mg/L. These absolute translations provide an interpretable scale for the magnitude of group differences: the moderate symptom category (10–14) differed from the reference by approximately +0.20 mg/L on the geometric mean scale, whereas the 15–19 category differed by nearly +0.89 mg/L. Importantly, these are differences in adjusted geometric means and do not imply that depressive symptoms cause changes in hs-CRP.

Although adjusted geometric means in all categories were below 3 mg/L, the binary outcome (hs-CRP > 3 mg/L) captures the upper tail of the hs-CRP distribution rather than the central tendency. Therefore, even modest shifts in geometric means can correspond to meaningful differences in the proportion exceeding a clinical threshold, depending on the shape and spread of the underlying distribution.

### 3.4. Associations Between Depressive Symptoms and hs-CRP (Continuous and Binary Outcomes)

Primary association estimates for PHQ-9 categories are presented in Table 4. For continuous hs-CRP, effect estimates are reported as geometric mean ratios (GMR) relative to PHQ-9 0–4. For elevated inflammation (hs-CRP > 3 mg/L), effect estimates are reported as prevalence ratios (PR), again relative to PHQ-9 0–4. Both outcomes are derived from survey-weighted models using the core covariate adjustment set.

In the mild symptom category (PHQ-9 5–9), adjusted hs-CRP was modestly higher than the reference category, with a GMR of 1.10 (95% CI 1.00–1.20). The corresponding PR for hs-CRP > 3 mg/L was 1.15 (95% CI 1.04–1.28), indicating a higher prevalence of elevated inflammation in this group. This contrast illustrates how a relatively small upward shift in central tendency can be accompanied by a detectable difference in a threshold-based outcome when a substantial fraction of the population lies near the threshold.

For the pre-specified primary contrast (PHQ-9 10–14 vs. 0–4), the adjusted GMR was 1.14 (95% CI 0.96–1.35) and the PR was 1.15 (95% CI 0.95–1.39). Point estimates were above the null for both outcomes, but the confidence intervals encompassed the null value. Therefore, the data are compatible with a modest positive association, as well as with no association, for this moderate symptom stratum under the core adjustment set.

The largest associations were observed for PHQ-9 15–19, with a GMR of 1.62 (95% CI 1.20–2.19) and a PR of 1.49 (95% CI 1.15–1.92). These estimates indicate substantially higher adjusted hs-CRP levels and a higher prevalence of hs-CRP > 3 mg/L relative to minimal symptoms. For PHQ-9 20–27, the adjusted GMR remained above the null (1.34; 95% CI 1.08–1.67), while the PR for hs-CRP > 3 mg/L was 1.13 (95% CI 0.82–1.57). The discrepancy between continuous and binary outcomes in the highest symptom stratum is consistent with reduced precision and potential threshold effects, and it underscores that inferences for hs-CRP > 3 mg/L in this stratum should be weighted towards uncertainty rather than point estimates.

In a clinically relevant dichotomy (PHQ-9 ≥ 10 vs. <10), depressive symptoms were associated with higher hs-CRP (GMR 1.24, 95% CI 1.07–1.43) and a higher prevalence of hs-CRP > 3 mg/L (PR 1.19, 95% CI 1.01–1.39) after adjustment for the core covariate set. In exploratory interaction models, there was no strong evidence that associations differed by sex (Wald p_interaction = 0.59 for log(hs-CRP) and 0.59 for hs-CRP > 3 mg/L). Similarly, interaction models by broad age group (20–39, 40–59, ≥60) provided little evidence of effect modification (Wald p_interaction = 0.59 for log(hs-CRP) and 0.87 for hs-CRP > 3 mg/L).

### 3.5. Non-Linearity of the PHQ-9-hs-CRP Association (Spline Analysis)

**Figure 2:**
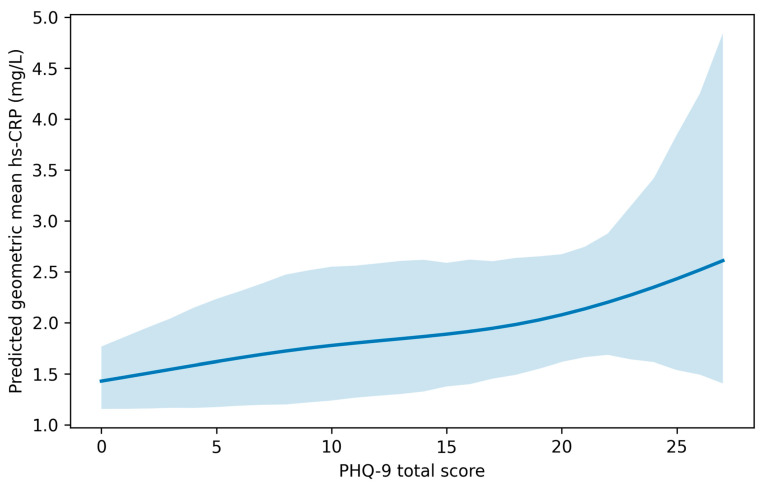
Spline-based predicted hs-CRP across the PHQ-9 score range. Note: Curve shows model-based predicted geometric mean hs-CRP (mg/L) across PHQ-9 total score from a survey-weighted log(hs-CRP) model with natural splines (4 df) for PHQ-9 and the core covariate set. Shaded bands indicate 95% confidence intervals. Predictions are evaluated at representative covariate values (median age and median PIR) and fixed categorical covariates (female; never smoker; race/ethnicity and education set to model reference levels).

To complement category-based estimates and explore functional form, we fitted an adjusted model using natural splines for PHQ-9 total score and generated model-based predictions of the geometric mean hs-CRP across PHQ-9 values. The resulting curve is displayed in Figure 2. This exploratory analysis supports interpretation of potential thresholds or regions where the association appears stronger.

Spline-based predictions suggested a gradual increase in predicted hs-CRP through the lower and moderate PHQ-9 ranges, followed by a steeper rise at higher PHQ-9 values. The widening confidence bands at higher PHQ-9 scores reflect sparse data in the upper symptom range and should be interpreted cautiously. Overall, the spline pattern was consistent with the category-based results, supporting a non-linear association in which elevations are more apparent at higher symptom severity.

### 3.6. Results of the Sensitivity Analyses

Sensitivity analyses for the primary contrast (PHQ-9 10–14 vs. 0–4) are presented in Table 5. Two pre-specified sensitivity dimensions were prioritised: exclusion of hs-CRP > 10 mg/L to reduce potential influence of acute inflammatory states, and addition of BMI to the core adjustment set to evaluate sensitivity to adiposity-related pathways. For both outcomes, we report the range of point estimates across specifications for the primary contrast.

Across specifications, the GMR ranged from 1.01 to 1.14, and the PR ranged from 1.05 to 1.15. Relative to the main-model point estimates (GMR 1.14; PR 1.15), exclusion of hs-CRP > 10 mg/L attenuated the GMR to 1.01 and the PR to 1.10, while additional adjustment for BMI attenuated the GMR to 1.01 and the PR to 1.05. These attenuations indicate that inferences for the moderate-symptom contrast are sensitive to handling of high hs-CRP values and to adiposity-related adjustment.

In practical terms, the sensitivity analyses indicate that the moderate symptom stratum provides limited evidence of an association after accounting for these alternative specifications, with confidence intervals still spanning the null. By contrast, the moderately severe symptom category (15–19) demonstrated larger and more precise main-model associations, suggesting that elevated inflammation may be more evident in this severity range under the current adjustment strategy.

In this survey-weighted NHANES analysis of adults, higher depressive symptom severity was associated with higher adjusted hs-CRP levels and, in several strata, a higher prevalence of hs-CRP > 3 mg/L relative to minimal symptoms. The clearest and most precise associations were observed for PHQ-9 15–19. The pre-specified primary contrast (PHQ-9 10–14 vs. 0–4) produced point estimates above the null but with confidence intervals spanning the null, and sensitivity analyses attenuated estimates towards the null. Exploratory spline analyses supported a non-linear pattern with steeper increases in predicted hs-CRP at higher PHQ-9 scores, consistent with the category-based findings.

## 4. Discussion

In this nationally representative analysis of U.S. adults from NHANES 2015–2018, higher depressive symptom severity was associated with higher hs-CRP concentrations and a higher prevalence of low-grade systemic inflammation. In the pre-specified primary contrast (PHQ-9 10–14 vs. 0–4), estimates were modest and imprecise (GMR 1.14 (0.96–1.35); PR 1.15 (0.95–1.39)), whereas associations were larger for PHQ-9 15–19 (GMR 1.62 (1.20–2.19); PR 1.49 (1.15–1.92)).

All primary estimates accounted for the complex NHANES sampling design and adjusted for a core confounder set (age, sex, race/ethnicity, education, poverty-income ratio, and smoking). In absolute terms, adjusted geometric mean hs-CRP was 1.43 mg/L (1.21–1.70) in the reference group (PHQ-9 0–4) and 1.63 mg/L (1.29–2.08) in the moderate-symptom group (PHQ-9 10–14), corresponding to a +0.20 mg/L difference. Using a clinically relevant dichotomy (PHQ-9 ≥ 10 vs. <10), depressive symptoms were associated with higher hs-CRP on average (GMR 1.24 (1.07–1.43)) and a higher prevalence of hs-CRP > 3 mg/L (PR 1.19 (1.01–1.39)).

Exploratory interaction models provided little evidence that associations differed by sex (p_interaction = 0.59 for log(hs-CRP) and 0.59 for hs-CRP > 3 mg/L). Sex-stratified associations were directionally similar and imprecise in both groups.

Sensitivity analyses indicated that inference for the primary contrast depended on handling of very high hs-CRP values and on adjustment for adiposity. Excluding hs-CRP > 10 mg/L attenuated the primary contrast (GMR 1.01 (0.88–1.17); PR 1.10 (0.86–1.42)), consistent with some influence of acute-phase responses or intercurrent infection. Additional adjustment for BMI substantially attenuated the contrast (GMR 1.01 (0.88–1.16); PR 1.05 (0.88–1.25)), which may reflect confounding by adiposity, mediation through adiposity-related pathways, or over-adjustment depending on the causal structure. Collectively, these results suggest that any depression-hs-CRP association in population data may be modest and partly explained by adiposity-related processes. Accordingly, the +BMI models should be interpreted as sensitivity specifications for adiposity-related structure (confounding, mediation, or both) rather than definitive confounder control.

### 4.1. Comparison with Prior Evidence

Our estimates are compatible with the established literature linking depression to systemic inflammation, while also illustrating that effect sizes in population-representative data are typically small-to-moderate once confounding and measurement issues are addressed. A widely cited meta-analysis of 30 studies reported a small positive association between depression and circulating CRP (effect size d ≈ 0.15), alongside stronger associations for IL-6 and IL-1β [32]. In a cumulative meta-analysis focusing on major depressive disorder, Haapakoski and colleagues reported elevated concentrations of IL-6, IL-1β, TNF-α and CRP in cases relative to controls, with a larger pooled effect for CRP (d ≈ 0.47) than observed in community samples that include milder symptom profiles [33]. Longitudinal evidence suggests that inflammation may precede subsequent depressive symptoms: Valkanova et al. synthesised longitudinal studies and found that elevated baseline CRP and IL-6 were associated with a small but statistically significant increased risk of later depressive symptoms, with the association persisting after adjustment for age and other covariates (adjusted r ≈ 0.046 for CRP) [34]. Prospective cohort analyses similarly indicate that higher CRP categories predict later depression and related distress measures, although effect sizes remain modest and are sensitive to adjustment and outcome definitions [35].

Our non-linear pattern-particularly the larger elevation in hs-CRP among individuals with PHQ-9 15–19-may be consistent with work suggesting that inflammation is more prominent in a subset of depression phenotypes and at higher symptom burdens. A systematic review and meta-analysis of CRP distributions in depression estimated that approximately 27% of individuals with depression have CRP levels > 3 mg/L and about 58% have CRP levels > 1 mg/L, supporting the concept of an “inflamed depression” subgroup rather than a uniform shift across all cases [36]. In that context, our finding that the prevalence of hs-CRP > 3 mg/L is higher in the moderately severe category (PR 1.49 (1.15–1.92)) may reflect enrichment of individuals with inflammatory comorbidity, cardiometabolic risk, or behavioural correlates of inflammation in that severity stratum.

Evidence from contemporary population-based studies also broadly converges with our observations. In a recent NHANES-based analysis (2017–2020), higher hs-CRP was associated with higher odds of depressive symptoms, with an adjusted OR of 1.10 (95% CI 1.01–1.21) per unit increase in hs-CRP and an adjusted OR of 1.39 (1.01–1.92) for the highest versus lowest hs-CRP quartile [37]. While that study reverses the exposure-outcome framing relative to ours, the concordant direction reinforces the plausibility of a shared inflammatory-depressive symptom nexus in nationally representative U.S. data. At the same time, observational studies vary in their handling of acute elevations, adiposity, medication use, and comorbid conditions-factors that can meaningfully influence effect sizes and, in some settings, the apparent directionality of associations [38,39].

### 4.2. Biological and Behavioural Mechanisms

Several mechanisms could underlie an association between depressive symptoms and elevated hs-CRP in population data. First, proinflammatory cytokines (notably IL-6 and TNF-α) stimulate hepatic CRP production, and meta-analytic evidence supports higher concentrations of these cytokines in major depression [40]. Inflammation may influence neurobiological pathways relevant to mood regulation, including monoaminergic signalling, glutamatergic transmission, and neuroendocrine stress systems; in turn, stress-related neuroimmune activation may contribute to symptom persistence or treatment resistance in a subset of patients [41,42]. Second, depressive symptoms are correlated with behaviours and exposures that elevate CRP, such as smoking, lower physical activity, sleep disturbance, and dietary patterns; even with adjustment, residual confounding by these or related factors is plausible. Third, adiposity and insulin resistance can drive low-grade inflammation and are also associated with depressive symptoms; prospective data from occupational cohorts indicate that inflammatory biomarkers can predict future depressive symptoms, but associations are often attenuated after accounting for health behaviours and metabolic risk profiles [39]. Importantly, IL-6 and CRP are not unambiguous markers of chronic inflammatory tone; they may also reflect regulatory or tissue-repair processes, acute exposures, and pleiotropic immune functions, which complicates a simplistic interpretation of “higher CRP = more inflammation” in observational research [43]. This nuance is relevant to our sensitivity findings, where exclusion of hs-CRP > 10 mg/L substantially weakened associations, consistent with acute or transient processes influencing hs-CRP variability and its relationship with mood symptoms.

### 4.3. Clinical and Public Health Implications

The magnitude of association observed for moderate symptoms (PHQ-9 10–14) is modest, and the confidence intervals include the null, so the results do not support using hs-CRP as a standalone marker of depressive symptom burden in general population screening. Nevertheless, the stronger association at PHQ-9 15–19 and the higher prevalence of hs-CRP > 3 mg/L in that category (PR 1.49 (1.15–1.92)) suggest that inflammatory status may be clinically relevant for a subset of individuals with higher symptom severity. This aligns with the emerging literature on stratified or precision approaches to depression treatment that consider inflammatory biomarkers as potential effect modifiers. Randomised evidence indicates that anti-inflammatory interventions can reduce depressive symptoms in some contexts: a meta-analysis of randomised clinical trials reported improvements in depressive symptoms with anti-inflammatory agents, although many trials were at high risk of bias and effects varied across drug classes and populations [44,45]. Likewise, a meta-analysis focused on anti-cytokine therapies in chronic inflammatory conditions found evidence of antidepressant activity, raising the possibility that cytokine pathway modulation can influence mood symptoms under certain biological conditions [46]. A proof-of-concept randomised trial of a TNF antagonist in treatment-resistant depression suggested differential response by baseline inflammatory markers, including hs-CRP, reinforcing the hypothesis that inflammatory status may help identify patients more likely to benefit from immunomodulatory strategies [47]. These findings should be interpreted cautiously, given heterogeneity of trial designs, potential adverse effects (including infection risk), and the fact that most evidence concerns clinical populations rather than community samples [45].

From a public health perspective, our results support the view that depressive symptoms and inflammation co-occur at the population level and that shared upstream determinants-such as socioeconomic disadvantage, smoking, physical inactivity, and cardiometabolic conditions-may drive both. Therefore, interventions that improve behavioural and metabolic risk profiles may be relevant for both depressive symptoms and inflammatory burden, but this cross-sectional analysis cannot determine whether such interventions would reduce hs-CRP or depressive symptoms. The modest effect sizes observed for the primary contrast underscore that any translation into risk prediction or targeted screening should focus on carefully selected subgroups and on multiparameter risk profiles rather than a single biomarker.

### 4.4. Alternative Explanations, Causality, and Triangulation

Because this is a cross-sectional analysis, reverse causality and residual confounding cannot be ruled out. Inflammatory activity could contribute to depressive symptoms (as suggested by longitudinal meta-analyses [34] and prospective cohorts [35]), depressive symptoms could influence inflammatory tone through behavioural and neuroendocrine pathways [41,42], or both may be jointly influenced by unmeasured factors. Genetic triangulation studies provide mixed support for a direct causal role of CRP on depression. For example, a recent Mendelian randomisation study reported evidence that much of the association between CRP and depressive symptoms is confounded by BMI and related metabolic traits, with limited evidence for a direct causal effect of CRP on depression once BMI is accounted for [48]. Other longitudinal modelling approaches have also failed to identify strong bidirectional effects between CRP and depressive symptoms in some samples, suggesting that observed associations may be contingent on population characteristics, measurement frequency, and the degree of metabolic confounding [49]. Our sensitivity results-especially the attenuation after excluding hs-CRP > 10 mg/L and the modest range of estimates across robustness specifications-are consistent with a model in which a combination of confounding, acute-phase processes, and heterogeneity of depression phenotypes explains a considerable share of the observed association.

### 4.5. Strengths and Limitations

Key strengths include the use of a large, nationally representative dataset with complex survey weights, a validated measure of depressive symptoms [24], and hs-CRP assays with established analytic protocols [26]. The analytic plan was anchored to a prespecified adjustment set informed by a directed acyclic graph and included sensitivity checks designed to assess robustness to key modelling decisions. The results also provide clinically interpretable metrics on both continuous and dichotomised hs-CRP outcomes.

Several limitations merit emphasis. First, cross-sectional data preclude causal inference and temporal ordering; the results should be interpreted as associations. Second, hs-CRP is a non-specific biomarker subject to within-person variability and acute influences, and single measurements may misclassify chronic inflammatory status. Third, although we adjusted for a core confounder set, residual confounding by unmeasured or imperfectly measured factors (e.g., diet quality, sleep duration and disorders, antidepressant and anti-inflammatory medications, and comorbid inflammatory conditions) is possible. Fourth, NHANES 2015–2018 predates the COVID-19 pandemic, and depressive symptom burden and inflammatory profiles may have shifted subsequently; replication in more recent cycles and longitudinal cohorts would strengthen inference. Fifth, laboratory methods for hs-CRP changed across the two pooled cycles; while NHANES provides harmonised measures, some measurement heterogeneity may persist. Finally, precision was limited for high-symptom categories (e.g., PHQ-9 20–27) due to small unweighted counts and complex survey design, leading to wide confidence intervals. We explored heterogeneity by sex and broad age group via interaction models and found little evidence of effect modification, but other sources of heterogeneity (race/ethnicity, metabolic status) warrant careful prespecification in future work.

### 4.6. Certainty and Implications

Moderate for the direction of association between higher depressive symptom severity and higher hs-CRP in the general adult population; low-to-moderate for the magnitude of the primary contrast (PHQ-9 10–14 vs. 0–4) because confidence intervals include the null and estimates are sensitive to exclusion of hs-CRP > 10 mg/L.

Hs-CRP should not be used as a standalone proxy for depressive symptom severity in population screening; however, elevated hs-CRP among individuals with higher PHQ-9 scores may help motivate integrated cardiometabolic and mental health risk assessment in selected clinical contexts.

Programmes that target shared determinants (smoking, inactivity, metabolic risk) may influence both depressive symptom burden and inflammatory burden through common upstream pathways, although causal effects cannot be inferred from these cross-sectional data.

By reporting both relative (GMR/PR) and absolute (geometric mean differences) metrics in a nationally representative sample and explicitly testing the impact of excluding hs-CRP > 10 mg/L, this work clarifies the extent to which acute-phase elevations may influence depression-hs-CRP associations in survey data.

Higher depressive symptom severity was associated with higher hs-CRP and a higher prevalence of low-grade systemic inflammation, with the strongest association observed among adults with moderately severe symptoms. The findings support a co-occurrence model between depressive symptoms and systemic inflammation in the population, while highlighting that effect sizes are modest, non-linear across symptom categories, and sensitive to handling of high hs-CRP values. Future research should prioritise longitudinal designs, repeated biomarker measurements, and triangulation with genetic and experimental evidence to clarify directionality and to evaluate whether inflammation-informed prevention or treatment strategies may be beneficial for specific subgroups.

## 5. Conclusions

Using nationally representative survey-weighted data from U.S. adults, higher depressive symptom severity was associated with higher hs-CRP levels and, in several symptom strata, a higher prevalence of low-grade systemic inflammation after adjustment for a pre-specified core confounder set. The pre-specified primary contrast (PHQ-9 10–14 versus 0–4) yielded modest point estimates above the null for both continuous and dichotomised hs-CRP outcomes, but with uncertainty compatible with little or no association under the primary specification.

Across the full range of symptom categories, associations were not strictly monotonic, but the strongest and most precise elevations in hs-CRP were observed among participants with moderately severe symptoms (PHQ-9 15–19). Exploratory modelling of the PHQ-9 score continuum supported a non-linear pattern in which predicted hs-CRP rose more steeply at higher symptom levels, consistent with heterogeneity in inflammatory burden across depression severity.

Robustness checks indicated that inferences for the primary contrast were sensitive to handling of high hs-CRP values and to additional adjustment for adiposity, with attenuation towards the null under alternative specifications. These results support a co-occurrence model between depressive symptoms and systemic inflammation at the population level, while emphasising that effect sizes are generally modest and that careful specification and sensitivity analyses are essential when interpreting depression-hs-CRP associations in cross-sectional survey data.

## Figures and Tables

**Table 1:** Unweighted analytic sample size by PHQ-9 category.

PHQ-9 Category	*n*	% of Analytic Sample
0–4	6823	74.5%
5–9	1554	17.0%
10–14	494	5.4%
15–19	203	2.2%
20–27	90	1.0%

Note: Counts are unweighted. Categories follow standard PHQ-9 severity groupings: 0–4 (minimal), 5–9 (mild), 10–14 (moderate), 15–19 (moderately severe), and 20–27 (severe).

**Table 2:** Survey-weighted participant characteristics by PHQ-9 category.

Characteristic	PHQ-9 0–4	PHQ-9 5–9	PHQ-9 10–14	PHQ-9 15–19	PHQ-9 20–27
Continuous variables, mean (95% CI)					
Age, years	48.2 (47.2–49.2)	48.2 (46.7–49.8)	46.6 (44.6–48.7)	48.9 (45.6–52.2)	46.4 (41.0–51.8)
Poverty-income ratio (PIR)	3.24 (3.11–3.38)	2.62 (2.50–2.74)	2.39 (2.18–2.60)	2.09 (1.80–2.37)	1.71 (1.20–2.21)
Body mass index (BMI), kg/m^2^	29.4 (29.0–29.8)	30.4 (29.7–31.2)	30.9 (29.7–32.0)	31.9 (30.2–33.6)	29.8 (28.1–31.5)
Categorical variables, %					
Female	48.9%	58.2%	65.4%	57.6%	61.3%
Male	51.1%	41.8%	34.6%	42.4%	38.7%
Race/ethnicity: Mexican American	8.5%	9.2%	6.6%	8.0%	7.7%
Race/ethnicity: Other Hispanic	5.8%	6.2%	6.6%	7.4%	7.7%
Race/ethnicity: Non-Hispanic White	66.5%	65.8%	63.8%	67.8%	66.0%
Race/ethnicity: Non-Hispanic Black	10.0%	10.5%	11.6%	9.6%	6.8%
Race/ethnicity: Non-Hispanic Asian	5.5%	3.5%	3.0%	1.4%	2.0%
Race/ethnicity: Other/Multiracial	3.9%	4.8%	8.4%	5.8%	9.9%
Education: <High school	10.1%	14.2%	14.9%	24.1%	25.4%
Education: High school/GED	23.2%	28.3%	28.0%	22.6%	38.9%
Education: Some college/AA	31.3%	35.0%	35.4%	38.3%	26.3%
Education: College graduate+	35.4%	22.5%	21.5%	15.0%	9.4%
Smoking: Never	60.2%	46.8%	42.1%	31.2%	24.1%
Smoking: Former	25.8%	27.9%	25.3%	22.8%	23.3%
Smoking: Current	14.1%	25.3%	32.5%	46.0%	52.7%

Note: Percentages are survey-weighted within PHQ-9 categories; continuous variables are survey-weighted means (95% CI). Estimates are based on the complete-case core covariate sample (*n* = 8173); BMI estimates exclude missing BMI. Values may not sum to exactly 100% due to rounding.

**Table 3:** Adjusted geometric mean hs-CRP by PHQ-9 category.

PHQ-9 Category	Adjusted Geometric Mean hs-CRP, mg/L (95% CI)
0–4	1.43 (1.21–1.70)
5–9	1.58 (1.29–1.92)
10–14	1.63 (1.29–2.08)
15–19	2.32 (1.59–3.37)
20–27	1.92 (1.42–2.60)

Note: Adjusted estimates are from the survey-weighted log(hs-CRP) model with the core covariate set (complete-case *n* = 8173). Adjusted geometric means correspond to exponentiated model-based estimated marginal means for each PHQ-9 category; 95% confidence intervals are computed on the log scale and back-transformed.

**Table 4:** Adjusted associations of depressive symptoms with hs-CRP (categorical PHQ-9 and PHQ-9 ≥ 10 vs. <10).

Contrast (vs. PHQ-9 0–4)	GMR for hs-CRP (95% CI)	PR for hs-CRP > 3 mg/L (95% CI)
5–9 vs. 0–4	1.10 (1.00–1.20)	1.15 (1.04–1.28)
10–14 vs. 0–4	1.14 (0.96–1.35)	1.15 (0.95–1.39)
15–19 vs. 0–4	1.62 (1.20–2.19)	1.49 (1.15–1.92)
20–27 vs. 0–4	1.34 (1.08–1.67)	1.13 (0.82–1.57)
PHQ-9 ≥ 10 vs. < 10	1.24 (1.07–1.43)	1.19 (1.01–1.39)

Note: GMR = geometric mean ratio from the survey-weighted log(hs-CRP) model. PR = prevalence ratio from the survey-weighted log-link model for hs-CRP > 3 mg/L. All models adjust for age, sex, race/ethnicity, education, PIR, and smoking. The PHQ-9 ≥ 10 vs. <10 row is derived from a separate model with dichotomised PHQ-9 for clinical interpretability.

**Table 5:** Sensitivity analyses for the primary contrast (PHQ-9 10–14 vs. 0–4).

Model Specification	Estimate (95% CI)
Main: GMR (log hs-CRP)	1.14 (0.96–1.35)
Main: PR (hs-CRP > 3 mg/L)	1.15 (0.95–1.39)
Sensitivity: GMR excl hs-CRP > 10 mg/L	1.01 (0.88–1.17)
Sensitivity: PR excl hs-CRP > 10 mg/L	1.10 (0.86–1.42)
Sensitivity: GMR + BMI	1.01 (0.88–1.16)
Sensitivity: PR + BMI	1.05 (0.88–1.25)

Note: Main models use the core covariate set. Sensitivity models either exclude hs-CRP > 10 mg/L or add BMI to the core covariate set. Excluding hs-CRP > 10 mg/L removed 721/8173 participants (8.8%) (*n* = 7452). BMI-adjusted models were estimated among participants with non-missing BMI (*n* = 8099). Estimates are GMR for continuous hs-CRP (log model) or PR for hs-CRP > 3 mg/L (log-link model).

## Data Availability

The datasets analysed in this study are publicly available from the National Health and Nutrition Examination Survey (NHANES) repository: https://wwwn.cdc.gov/nchs/nhanes/ (accessed on 26 January 2026).

## Abbreviations

The following abbreviations are used in this manuscript:

Abbreviation	Full Term (British English)
ACC/AHA	American College of Cardiology/American Heart Association
BMI	Body Mass Index
CI	Confidence Interval
CRP	C-Reactive Protein
DAG	Directed Acyclic Graph
ERB	Ethics Review Board
GMR	Geometric Mean Ratio
hs-CRP	High-Sensitivity C-Reactive Protein
IL	Interleukin
MEC	Mobile Examination Center
NCHS	National Center for Health Statistics
NHANES	National Health and Nutrition Examination Survey
OR	Odds Ratio
PHQ-9	Patient Health Questionnaire-9
PIR	Poverty-Income Ratio
PR	Prevalence Ratio
SDMVPSU	Sampling Design Variable-Primary Sampling Unit
SDMVSTRA	Sampling Design Variable-Stratum
TNF	Tumour Necrosis Factor
UK	United Kingdom
US	United States
WHO	World Health Organization
